# Acute lymphoblastic leukemia relapsing after first-line pediatric-inspired therapy: a retrospective GRAALL study

**DOI:** 10.1038/bcj.2016.111

**Published:** 2016-12-09

**Authors:** A Desjonquères, P Chevallier, X Thomas, F Huguet, T Leguay, M Bernard, J-O Bay, E Tavernier, A Charbonnier, F Isnard, M Hunault, P Turlure, M Renaud, J-N Bastié, C Himberlin, S Lepretre, B Lioure, V Lhéritier, V Asnafi, K Beldjord, M Lafage-Pochitaloff, M C Béné, N Ifrah, H Dombret

**Affiliations:** 1 Hematology Department, University Hospital, Nantes, France; 2 Hematology Department, University Hospital, Lyon, France; 3 Hematology Department, University Hospital, Toulouse, France; 4 Hematology Department, University Hospital, Bordeaux, France; 5 Hematology Department, University Hospital, Rennes, France; 6 Hematology Department, University Hospital, Clermont-Ferrand, France; 7 Hematology Department, University Hospital, Saint-Etienne, France; 8 Hematology Department, Institut Paoli-Calmette, Marseille, France; 9 Hematology Department, Hopital St-Antoine, Assistance Publique - Hôpitaux de Paris (AP-HP), Paris, France; 10 Hematology Department, University Hospital & University, INSERM U892/CNRS6299, Angers, France; 11 Hematology Department, University Hospital, Limoges, France; 12 Hematology Department, University Hospital, Poitiers, France; 13 Hematology Department, University Hospital, Dijon, France; 14 Hematology Department, University Hospital, Reims, France; 15 Hematology Department, Centre Henri Becquerel, Rouen, France; 16 Hematology Department, University Hospital, Strasbourg, France; 17 GRAALL, Lyon, France; 18 Université Paris Descartes Sorbonne Cité, Institut Necker-Enfants Malades (INEM), Institut National de Recherche Médicale (INSERM) U1151, and Laboratory of Onco-Hematology, Assistance Publique-Hôpitaux de Paris (AP-HP), Hôpital Necker Enfants-Malades, Paris, France; 19 Department of Molecular Biology, University Hospital Saint Louis (AP-HP), Paris, France; 20 Département de Génétique, Aix Marseille University, APHM, Hopital Timone, Marseille, France; 21 Hematology Biology, University Hospital, Nantes, France; 22 Hematology Department, University Hospital Saint-Louis, AP-HP, University Paris Diderot, Paris, France

## Abstract

The outcome of adult patients with Philadelphia chromosome-negative acute lymphoblastic leukemia (Ph− ALL) relapsing after pediatric-inspired front-line therapy is ill known. Here 229 relapsing Ph− ALL younger adults (18–63 years) treated within the Group for Research on Adult Acute Lymphoblastic Leukemia (GRAALL)-2003/-2005 trials were considered. Salvage regimens consisted of potentially curative therapies in 194 cases, low-intensity therapies in 21, allogeneic stem cell transplant (allo-SCT) in 6 and best supportive care in 8. Overall, 77 patients received allo-SCT after relapse. The median follow-up was 3.1 years. A second complete remission (CR2) was achieved in 121 patients (53%). In multivariate analysis, only younger age <45 years (*P*=0.008) and CR1 duration ⩾18 months (*P*=0.009) predicted CR2. Overall survival (OS) at 2 and 5 years was 19.3% (14–24%) and 13.3% (8–18%), respectively. In CR2 patients, disease-free survival (DFS) at 2 and 5 years was 29.0% (21–38%) and 25% (17–33%). In multivariate analysis, CR1 duration ⩾18 months and allo-SCT after relapse were associated with longer DFS (*P*<0.009 and *P*=0.004, respectively) and longer OS (*P*=0.004 and *P*<0.0001, respectively). In conclusion, although younger adults relapsing after pediatric-inspired ALL therapies retain a poor outcome, some of them may be cured if CR1 duration ⩾18 months and if allo-SCT can be performed in CR2. New therapies are definitely needed for these patients.

## Introduction

The prognosis of adult patients with relapsed Philadelphia-negative (Ph−) acute lymphoblastic leukemia (ALL) is dismal.^1, 2, 3, 4, 5^ A frequent option in such circumstances is to obtain a new complete remission (CR) as a bridge to perform allogeneic stem cell transplantation (allo-SCT), which is the best prospect for cure.^5^ This strategy mainly applies to younger and fit patients who can receive aggressive salvage regimens. Second CR (CR2) rates in such patients have been shown to range between 30 and 45% and median survival between 6 and 9 months only.^1, 2, 3, 4, 5, 6, 7^ These data are mostly issued from studies incorporating patients treated before the era of pediatric-inspired strategies. The latter have yielded significant advances in this group of patients as demonstrated by the results of the two Group for Research on Adult Acute Lymphoblastic Leukemia (GRAALL)-2003 and -2005 trials.^8, 9, 10, 11, 12, 13, 14^ The GRAALL-2005 trial only differed from the 2003 trial by the addition of a randomized evaluation of hyperfractionated cyclophosphamide during induction and late intensification and by the randomization of rituximab addition during all phases of therapy in CD20^+^ B-cell precursor (BCP) ALL patients.^14^ High-risk patients^8, 12^ were candidate for allo-SCT in CR1.

The outcome of patients who relapsed after specific pediatric-like protocols in first-line therapy has to be further analyzed. The hypothesis was that as pediatric-based regimens have significantly improved survival, this might lead to a selection of patients with a more refractory disease/subclone. Here we retrospectively describe such results from a cohort of patients with relapsed Ph− ALL initially treated in the GRAALL-2003 and -2005 trials.

## Patients and methods

### Patients

Between 2003 and 2011, 955 younger adults with *de novo* Ph− ALL aged between 15 and 59 years from 70 French, Belgian and Swiss centers were treated within the multicenter prospective French GRAALL-2003 (*N*=225, period: 2003–2005) and GRAALL-2005 (*N*=730, period: 2006–2011) trials (clinicaltrials.gov, nos. NCT00222027 and NCT00327678, respectively). Of these 955 patients, 860 (548 BCP-ALL and 312 T-cell ALL (T-ALL)) reached CR1 and 311 further received allo-SCT in first CR (204 BCP-ALL and 107 T-ALL).^13^ Overall, 264 patients (30%) relapsed, including 58 after allo-SCT. For the purpose of this study, postrelapse information was collected retrospectively. First relapse and its issue was part of the information to be collected in the e-case report forms of the trials. Based on an extraction of these events, additional necessary information was retrieved from the patients' files.

### Genetic/molecular status at diagnosis

Patients were assigned to risk groups according to definitions applied to previous GRAALL trials.^8, 12^ Some relapsed patients were informative for their genetic/molecular status at diagnosis according to data published by the GRAALL^11, 12^ *MLL* (*KMT2A*) gene rearrangements and *IKZF1* gene deletion, for BCP-ALL, and *NOTCH1/FBXW7/RAS/PTEN* gene mutations/deletions, for T-ALL, were thus considered as potential prognostic factors. There were no analyses of minimal residual disease levels in CR2 patients.

### Statistical analyses

The primary objective of the study was to assess the outcome in younger Ph− ALL patients relapsing after having being treated in first-line therapy as part of the GRAALL-2003/-2005 trials. This was evaluated by assessing the CR2 rate as well as overall (OS) and disease-free survivals (DFS). Secondary objectives were to determine prognostic factors for CR2, DFS and OS.

CR2 was defined by a neutrophil count >1.0 × 10^9^/, a platelet count >100 × 10^9^/l and bone marrow blasts <5% while all extramedullary disease had resolved. Relapse after CR2 was defined as the reappearance of leukemic cells in the bone marrow or extramedullary. Patient characteristics and CR rates were compared using Fisher's exact test. Comparisons of medians were performed using the Mann–Whitney *U*-test.

OS was defined as the time from the day of first relapse to death or last follow-up. DFS was defined from the date of CR2 to that of second relapse or death or last follow-up. Survival outcomes were not censored at allo-SCT. OS and DFS were estimated using the Kaplan–Meier method and then compared using the log-rank test.^15^

Characteristics considered for univariate analysis were age (⩽45 years), ALL lineage (B vs T), ALL risk classification (high vs standard),^8, 12^ CR1 duration (⩽18 months), prior allo-SCT, relapse type (central nervous system vs others), relapse treatment type (intensive vs non-intensive, allo-SCT after relapse or not) and response to salvage regimen (CR2 or not). To evaluate the prognostic value of CR2 achievement, a landmark period of 82 days (75th percentile of the achievement of CR2) was used. Some lineage-specific cytogenetic or molecular features present at diagnosis were also considered, including t(4;11)(q21;q23)/*MLL-*AF4(KMT2A*-AFF1*), low hypodiploidy/near triploidy and *IKZF1* gene deletion for BCP-ALL and complex karyotype (⩾5 anomalies) or *NOTCH1/FBXW7/RAS/PTEN* mutational status for T-ALL.

Postrelapse allo-SCT was analyzed as a time-dependent event using Mantel–Byar estimations.^16^

Factors associated with a *P*-value <0.10 on univariate analysis were included in the final logistic regression hazard model for multivariate analysis. Multivariable regressions were performed with the Cox model.^17^ Hazard ratios (HRs) are given with 95% confidence interval (CI).

STATA/SE 10.1 software (STATA, College Station, TX, USA) was used. All tests were two-sided, with a type I error at 5%.

## Results

### Patients

**Figure 1:**
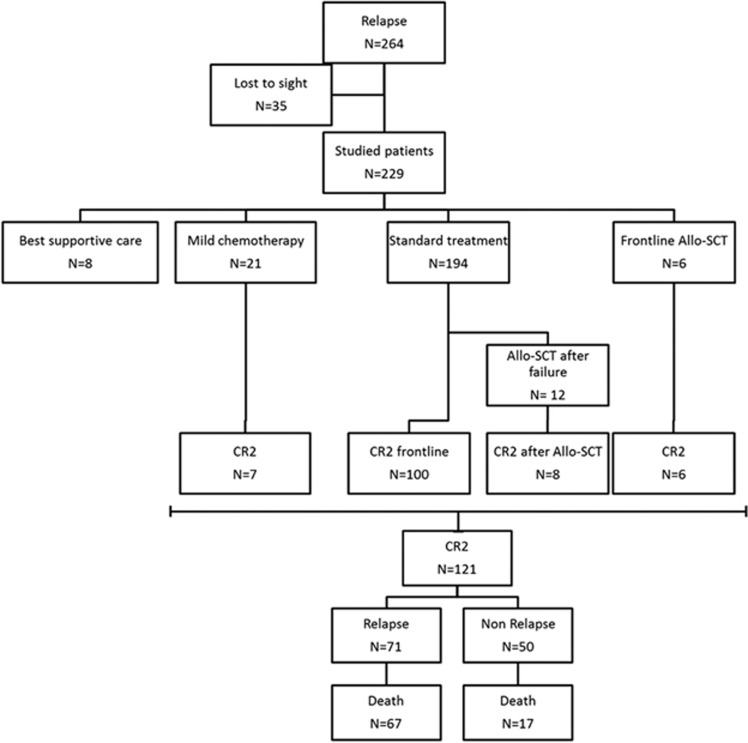
Flow chart.

Data were available for 229 of the 264 patients who relapsed after prior CR1 achievement. In this cohort, the median age at relapse was 35.7 years. Allo-SCT preceded relapse for 54 patients (24%) and the median duration of CR1 was 10 months (range, 0.5–74). One hundred and fifty-one patients (66%) had BCP-ALL and 165 patients (72%) carried high-risk characteristics^8, 12^ at diagnosis. The main site of relapse was bone marrow alone (*n*=181, 79%). Characteristics of the patients are given in Table 1. Details of the patients' evolution are given in the flow chart (Figure 1).

### Salvage regimens

All salvage regimens were permitted after relapse and details are given in Table 2. Most patients (*n*=194, 85%) were retreated intensively with a variety of reported regimens,^18, 19, 20, 21, 22, 23, 24, 25, 26, 27, 28, 29, 30, 31, 32, 33, 34, 35, 36, 37, 38, 39^ while 21 patients received less-intensive chemotherapy (mainly vincristine and corticosteroids), 6 upfront allo-SCT and 8 only best supportive care. The main novelties compared with previously reported salvage regimens were the use of second-generation purine analogs, such as clofarabine for BCP-ALL^18^ (34 patients) or nelarabine for T-ALL^19^ (12 patients), and a regimen based on L-asparaginase encapsulated in erythrocytes^21^ (12 patients). Only a few patients received immunotherapy with rituximab^40^ because of CD20 expression or gemtuzumab ozogamicin because of CD33 expression.^41^

Only the 215 patients who received an intensive (frontline allo-SCT excluded) or a less-intensive salvage regimen were considered for the analysis of prognostic factors of CR2. Only the 108 patients having obtained CR2 after intensive salvage regimen were considered for DFS analyses, and only the 221 who had received a treatment at relapse were considered for OS analyses.

### Factors predicting CR2, relapse and death after CR2

CR2 was achieved in 121 cases (53%), including 108 out of the 194 (56%) after intensive salvage regimen and 7 out of the 21 (33%) after a less-intensive salvage regimen. In addition, all of the six patients who received allo-SCT upfront as salvage regimen achieved CR2. Eight of the 108 patients in the intensive group achieved CR2 only after receiving allo-SCT as second-line salvage therapy. Risk stratification at diagnosis was not predictive of CR2 achievement (standard risk, 53% high risk, 50% unclassified, 58%). There was no statistically significant difference in the CR rates (51% for BCP-ALL vs 56% for T-ALL). Of the 14 T-ALL patients who received Nelarabine, 7 reached CR2. Of the 37 patients with central nervous system involvement at relapse, 26 reached CR2, 16 relapsed and 30 died. Among the 54 patients who had received AlloSCT before relapsing, 50% reached CR2.

In the univariate analysis, a younger age (<45 years; HR=0.48 (95% CI, 0.27–0.87); *P*=0.015) and a longer CR1 duration (>18 months; HR=0.45 (95% CI, 0.23–0.86); *P*=0.017) were associated with CR2 achievement. In BCP-ALL, the presence of t(4;11) (*n*=21) was also associated with failure to reach CR2 with only 20% of these patients reaching CR2.

In multivariate analysis, younger age (<45 years old; HR=0.44 (95% CI, 0.24–0.80); *P*=0.008) and longer CR1 duration (>18 months; HR=0.40 (95% CI, 0.20–0.80); *P*=0.009) were independently associated with CR2 achievement.

Among the 121 patients who reached CR2, 71 (59%) relapsed and 67 (94%) died after relapse. Of the 50 patients who did not relapse after CR2 (*n*=50), only 17 (34%) died. Of these 17 patients, 7 died of infectious complications, 6 of allo-SCT-related mortality, 2 of multivisceral organ failure and 2 of secondary hematological malignancy (1 AML, 1 NHL). Overall, 84 patients died (69%) after achieving CR2.

### Allo-SCT after first relapse

A total of 77 patients received allo-SCT after relapse: 6 (8%) upfront, 59 (77%) after reaching CR2 after intensive (*n*=55) or less-intensive (*n*=4) chemotherapy, and 12 (15%) after failure of the intensive salvage regimen. Among the 18 patients who received allo-SCT while with active disease, 14 (78%) achieved CR2 (6/6 upfront, and 8/12 refractory). The median time between relapse and allo-SCT was 111 days (range, 5–311). Seventeen patients received a reduced intensity conditioning (RIC) and 60 a myeloablative conditioning. For 26 patients, the donor was a matched relative, for 2 a haploidentical relative and for 40 a matched 10/10 or mismatched 9/10 unrelated donor. Nine patients received a cord-blood allo-SCT. Of the 77 patients who received allo-SCT, 13 received a RIC regimen (median age 42 years old, range 19–63) and 64 a myeloablative regimen (median age 28 years, range 18–60, *P*=0.003). Of the 13 RIC patients, 8 died, including 4 after second relapse, and another one relapsed but was still alive at last news. By comparison, of the 64 patients with myeloablative conditioning, 27 relapsed and 38 died. These differences were not statistically significant.

### DFS and factors predicting DFS

**Figure 2:**
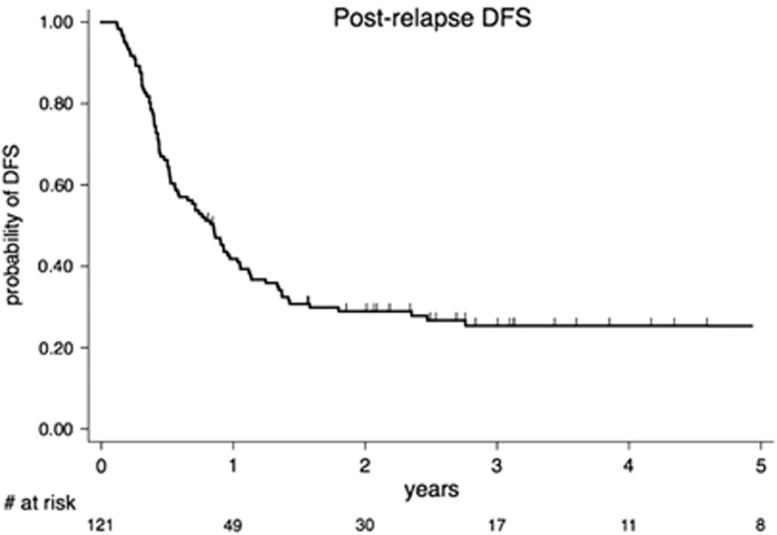
DFS for patients achieving second complete remission (CR2; *n*=121).

With a median DFS of 10.2 months (95% CI, 6.7–12.4), overall 1-, 2- and 5-year DFS for the 121 CR2 patients were 42% (95% CI, 0.32–0.50), 29% (95% CI, 0.21–0.37) and 25% (95% CI, 0.17–0.33), respectively (Figure 2).

In the univariate analysis, a significantly longer DFS was observed for patients with a CR1 duration ⩾18 months (HR=0.36 (95% CI, 0.21–0.62); *P*<0.0001) and for those who received allo-SCT after reaching CR2 (HR=0.45 (95% CI, 0.27–0.73); *P*=0.001). Multivariate analysis confirmed the independent favorable impact on DFS of longer CR1 duration (HR=0.41 (95% CI, 0.21–0.80); *P*<0.009) and of receiving allo-SCT after CR2 (HR=0.40 (95% CI, 0.22–0.75); *P*=0.004).

### OS and factors predicting OS

**Figure 3:**
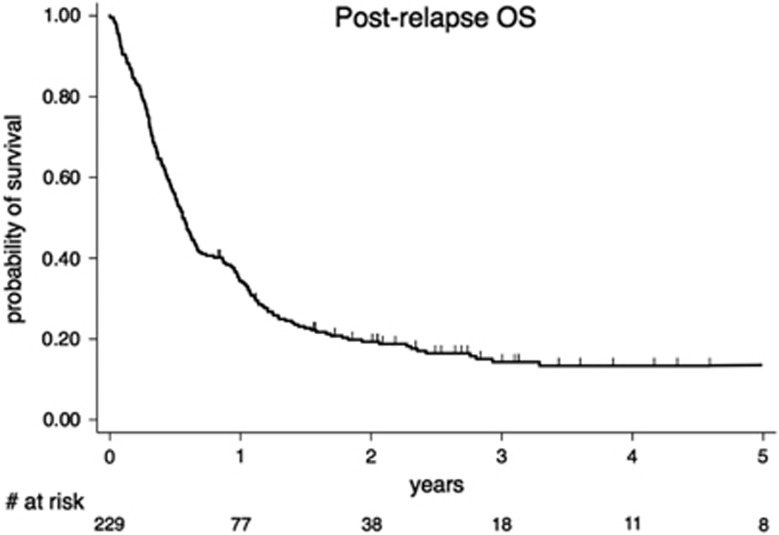
OS, (*N*=229).

The median follow-up for alive patients was 3.1 years. For the entire cohort, the median OS was 6.8 months (95% CI, 5.8–7.9) and 1-, 2- and 5-year OS were 34.4% (95% CI, 0.28–0.40), 19.3% (95% CI, 0.14–0.24) and 13.3% (95% CI, 0.08–0.18), respectively (Figure 3).

**Figure 4:**
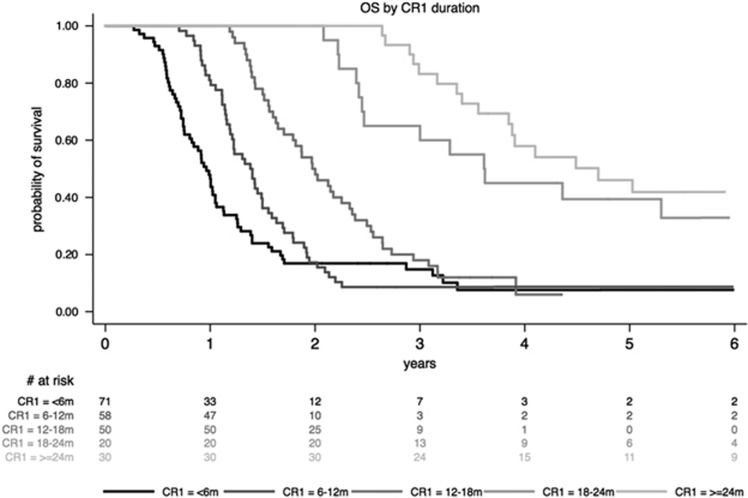
OS according to CR1 duration (m: months).

**Figure 5:**
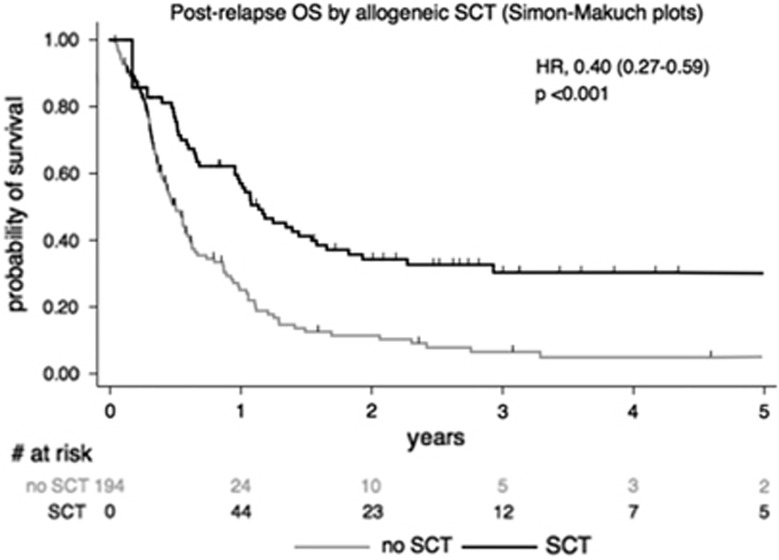
OS according to allo-SCT performed or not at relapse.

In the univariate analysis, factors associated with better OS were: younger age (⩽45 years; HR=0.68 (95% CI, 0.50–0.93); *P*=0.01), CR1 duration >18 months (HR=0.42 (95% CI, 0.29–0.63); *P*<0.0001; Figure 4), achievement of CR2 (HR=0.19 (95% CI, 0.16–0.27); *P*<0.001), and allo-SCT after first relapse (HR=0.40 (95% CI, 0.27–0.59); *P*<0.001; Figure 5). A worse OS was observed in BCP-ALL with t(4;11) (HR=2.13 (95% CI, 1.30–3.47); *P*=0.002) or low hypodiploidy/near triploidy (HR=2.96 (95% CI: 1.52–5.72); *P*=0.001) and in T-ALL with complex karyotype (HR=5.37 (95% CI, 2.18–13.23); *P*<0.001). Neither *IKZF1* gene deletion for BCP-ALL nor *NOTCH1/FBXW7/RAS/PTEN* mutational status for T-ALL did influence OS.

In the multivariate analysis, longer CR1 and allo-SCT after first relapse were the two factors that remained associated with prolonged OS (HR=0.53 (95% CI, 0.34–0.82); *P*=0.004; and HR=0.43 (95% CI, 0.27–0.66); *P*<0.0001, respectively).

## Discussion

The objective of this study was to assess the main outcomes (CR2 achievement, DFS, OS) of younger adults with Ph− ALL having relapsed after the pediatric-inspired GRAALL protocol. The main issue was to know whether any progress has been achieved in terms of survival regarding this relapsed population in recent years. Indeed, applying more intensive regimen could select, for relapse, patients with a more severe initial disease. This is partly supported by the lower rate of relapse observed in the GRAALL trials^8, 12^ compared with previous studies.^1, 2, 3, 4, 5^

Although the rate of CR2 (53%) was slightly higher, both the median DFS (10.2 months) and OS (6.8 months) remain similar to what has been previously reported.^1, 2, 3, 4, 5^ As in the past, a longer CR1 duration (>18 months) and allo-SCT after relapse (upfront or not) remain the two factors predicting a better outcome.^1, 2, 3, 4, 5, 6, 7^ These results suggest that no major breakthrough has been made in the recent years in the management of younger adults with Ph− ALL after relapse although increased survival has been obtained using pediatric-inspired therapy in first line.^8, 9, 10, 11, 12, 13, 14^ This also confirms that initial therapy does not really influence the outcome after relapse.^2, 3^

One striking point in the present study, confirming also previous reports,^1, 2, 3, 4, 5, 6, 7^ was the variety of salvage regimens used to treat patients at relapse. This emphasizes that still there is no standard-of-care treatment in this setting. The availability of new drugs such as clofarabine,^18^ nelarabine^19^ or L-asparaginase encapsulated in erythrocytes^21^ could have participated in the slightly higher CR2 rate (53%) observed in our series compared with previous reports.^1, 2, 3, 4, 5^ Yet, the longer CR1 duration possibly explained by the higher intensity of first-line treatment must also be taken into account. Emerging therapies, such as blinatumomab,^42^ inotuzumab ozogamicin,^43^ chimeric antigen receptor T cells^44^ or radio-labeled monoclonal antibodies,^45^ may soon increase the number of patients who will achieve CR2, in a non-intensive manner, which is of high interest in patients too frail to receive a standard salvage regimen.

The question remains of how to improve the results of these patients. Considering the factors predicting better OS, if we cannot influence such a parameter as CR1 duration, it remains that everything has probably still to be made to consider allo-SCT in all relapsing patients. The lower toxicity of RIC and the availability of alternative donors (cord blood or haplo-identical) may allow to proceed in time with allo-SCT for all these patients, even in case of comorbidities. However, it has to be recalled that patients who received allo-SCT after relapse in this series were in majority those who achieved CR2 (~80%) and that post-allo-SCT survival of patients with active disease at transplant (even if they finally achieved CR2) remains poor,^46, 47^ suggesting that upfront allo-SCT may not be an option in these circumstances. As a consequence, it appears crucial to obtain CR2, whatever the salvage regimen used, intensive or not. Interestingly, some prognostic genetic features observed at diagnosis, such as *IKZF1* gene deletion or *NOTCH1/FBXW7/RAS/PTEN* mutation status, were taken into account for their impact of postrelapse outcome. This has only been investigated in some pediatric publications.^48, 49, 50^ None of these molecular features had any impact in terms of postrelapse outcome in our study, perhaps because of the small number of informative cases. It may also be, although this was not investigated, that different clones were involved at relapse which did not express mutations or deletions found at diagnosis.^50^

Finally, the impact of minimal residual disease levels in adults reaching CR2 remains to be determined.The MRD level may help to discriminate patients who may benefit from additional therapy/maintenance, such as blinatumomab,^51^ before transplant.

In conclusion, younger adult patients relapsing after current ALL therapies still display a poor outcome. Yet, a minority of them may be cured, especially if CR1 duration has exceeded 18 months and if they are able to receive allo-SCT in CR2. New therapies have to be evaluated prospectively in these patients.

## Figures and Tables

**Table 1:** Patients' characteristics

*Patients*	N*=229*
Median follow-up (range), years	3.1 (2.7–4.15)
First-line trial, GRAALL-2003/-2005	65/164
Gender, male/female	148/81
Median age (range), years	35.6 (17.2–63.1)
<45 years/>45 years	166/63
Lineage, BCP-ALL/T-ALL	151/78
	
*High-risk cytogenetics at diagnosis*	
t(4;11)(q21;q23)/*MLL-AF4(KMT2A-AFF1)*	21
t(1;19)(q23;p13)/*E2A-PBX1 (TCF3-PBX1)*	6
Complex	18
Low hypodiploidy/near triploidy	10
	
*Molecular status at diagnosis*	
*IKZF1* gene deletion (BCP-ALL), yes/no	34/87
No *NOTCH1/FBXW7* mutation and/or *N/K-RAS* mutation and/or *PTEN* gene alteration (T-ALL), yes/no	42/56
	
Risk stratification at diagnosis, standard/high/unclassified^a^	45/65/19
	
*Response to treatment in first-line therapy*	
Corticoresistance,^b^ yes/no/not determined	62/165/2
Chemoresistance,^c^ yes/no/not determined	108/113/8
	
Salvage regimen needed to obtain CR1, yes/no	10/219
Allo-SCT in CR1	54
Duration of CR1, median, months (range)	10 (0.5–74)
<18 months/>18 months	179/50
	
*Site of relapse*	
Bone marrow	181
Central nervous system	19
Both	20
Other	9
Unknown	2

Abbreviations: ALL, acute lymphoblastic leukemia; allo-SCT, allogeneic stem cell transplantation; BCP, B-cell precursor; CR1, first complete remission; GRAALL, Group for Research on Adult Acute Lymphoblastic Leukemia; T-ALL, T-cell acute lymphoblastic leukemia.

a High-risk factors were a WBC count of ⩾30 × 10^9^/l for B-lineage ALL, clinical and/or morphological CNS involvement, t(4;11) and/or *MLL-AF4* fusion transcript, t(1;19) and/or *E2A-PBX1* fusion transcript, low hypodiploidy and/or near triploidy.^8^

b Poor peripheral blood blast clearance after the corticosteroid prephase.

c Poor bone marrow blast clearance after one additional week of induction chemotherapy.

**Table 2:** Salvage regimens

*Total patients*	N*=229*
Best supportive care	*N*=8
Upfront allo-SCT	*N*=6
	
*Less-intensive chemotherapy*	*N*=21
Vincristine/corticosteroids	8
Vincristine/low-dose methotrexate	4
Vincristine/6-mercaptopurine	3
6-Mercaptopurine+low-dose methotrexate	3
Intrathecal therapy (liposomal Ara-C)^18^	3
	
Intensive chemotherapy	*N*=194
	
*Newer regimens*	*N=60*
Clofarabine based^19^	34
Vandevol	27
Endevol	2
Other combinations	5
Nelarabine based^20^	12
L-asparaginase encapsulated in erythrocyte based^21^	12
Rapamycin based^22^	2
	
*Older regimens*	*N=134*
Pediatric like	
Vanda^23^	19
Cooprall^24^	6
Fralle-93^25^	1
Hyper-CVAD^26^	27
Anthracycline based	
Idarubicine–Ara-C^27^	27
Flag-Idarubicine^28^	2
Idarubicine–etoposide^29^	1
VAD^30^	5
Idarubicine/vincristine/L-asparaginase^31^	2
Daunorubicine/Ara-C+Cyclophosphamide^32^	4
Mitoxantrone/Ara-C (HAM)^33^	10
Mitoxantrone/Ara-C/etoposide/corticosteroid (PAME)^34^	1
Amsacrine/Ara-C^35^	1
Methotrexate based	
High-dose methotrexate+Ara-C^36^	6
Copadem^37^	2
GRAALL-2005 re-induction^12^	5
LALA-94 re-induction^38^	1
Capizzi re-induction^39^	4
Miscellaneous (etoposide/DEX, Ara-C/DEX, Ara-C/GO^41^)	4 (2,1,1)
Unknown	5

Abbreviations: allo-SCT, allogeneic stem cell transplantation; DEX, dexamethasone; GO, gemtuzumab ozogamicin; GRAALL, Group for Research on Adult Acute Lymphoblastic Leukemia.
